# Twin Growth Discordance and Risk of Postpartum Hemorrhage: A Retrospective Cohort Study

**DOI:** 10.3389/fmed.2022.876411

**Published:** 2022-05-25

**Authors:** Xiuhong Cao, Ye Luo, Shuangqiong Zhou, Qingsong Zhao, Xuewei Qin, Zhiqiang Liu, Zhendong Xu

**Affiliations:** ^1^Department of Anesthesiology, Shanghai First Maternity and Infant Hospital, School of Medicine, Tongji University, Shanghai, China; ^2^Department of Research and Education, Shanghai First Maternity and Infant Hospital, School of Medicine, Tongji University, Shanghai, China; ^3^Department of Obstetrics, Shanghai First Maternity and Infant Hospital, School of Medicine, Tongji University, Shanghai, China

**Keywords:** chorionicity, cesarean section, fetal growth restriction, growth discordance, postpartum hemorrhage, twin pregnancy

## Abstract

**Background:**

In recent years, the incidence of postpartum hemorrhage has increased globally. Multiple pregnancies and cesarean sections are well-known risk factors for postpartum hemorrhage. No studies have evaluated the associations between fetal growth anomalies and postpartum hemorrhage in women with twin pregnancies undergoing cesarean section. This study aimed to identify the relationship between fetal growth anomalies and postpartum hemorrhage in women with twin pregnancies undergoing cesarean section.

**Methods:**

This retrospective single-center study included 3,180 women with twin pregnancies at a tertiary hospital between August 2013 and July 2020. Singleton reference charts were used to assess fetal growth restriction at birth. Discordant growth was defined as an intertwin birth weight difference of ≥20%. Logistic regression analyses were used to evaluate the association between fetal growth anomalies and postpartum hemorrhage. Additionally, sensitivity analysis of abnormal placenta and stratification by twin chorionicity were conducted.

**Results:**

The overall incidence of postpartum hemorrhage was 4.3%. Twin growth discordance, especially with fetal growth restriction, was associated with an increased risk of postpartum hemorrhage (adjusted odds ratio [AOR] = 1.62, 95% confidence interval [CI], 1.05–2.51, *P* = 0.031; AOR = 1.71; 95% CI, 1.08–2.70, *P* = 0.022; AOR = 1.98, 95% CI, 1.21–3.25, *P* = 0.006, respectively). After stratification, this relationship persisted in dichorionic twins (OR = 1.71, 95% CI, 1.04–2.82, *P* = 0.036; OR = 1.90, 95% CI, 1.13–3.21, *P* = 0.016; OR = 2.48, 95% CI, 1.41–4.38, *P* = 0.002, respectively). However, no significant association was observed in monochorionic twin pregnancies.

**Conclusion:**

Growth discordance, especially complicated by fetal growth restriction, was associated with an increased risk of postpartum hemorrhage in women with twin pregnancies undergoing cesarean section, and was more evident in patients with dichorionic twins.

## Introduction

In recent years, the number and rate of multiple gestations, especially twin gestations, have risen dramatically due to advanced maternal age and the rapid development of assisted reproductive technology (ART) (1, 2). Twin pregnancies are more commonly affected by abnormal fetal growth, as the human uterus has a weak ability to meet the needs of more than one fetus. The most common abnormal fetal growth in twin pregnancies is fetal growth restriction (FGR) (3–6). In twin pregnancies, the incidence of FGR, depending on the definition of growth restriction, ranges from 16 to 48% (4). Even without FGR, twin pregnancies may be complicated by twin growth discordance, which is defined as a birth weight difference of ≥20% (7). Both FGR and twin growth discordance are known risk factors for adverse perinatal outcomes (8–15).

Postpartum hemorrhage (PPH) is one of the most common adverse perinatal outcomes in twin pregnancies. Postpartum hemorrhage is the leading cause of maternal death and poses critical health care challenges globally (16); therefore, it is crucial to identify pregnant women who are at a higher risk of PPH and implement early preventive strategies. Recently, factors including advanced maternal age, placenta previa, placental abruption, gestational hypertension, previous cesarean delivery, and fetal macrosomia have been reported to be associated with PPH (17–25). However, no study has investigated the effects of abnormal fetal growth on PPH.

This study aimed to explore the associations between twin pregnancies complicated by FGR or twin growth discordance and the risk of PPH in a retrospective twin birth cohort study conducted in Shanghai, China. Additionally, we investigated whether twin chorionicity (dichorionic and monochorionic) altered the association between abnormal fetal growth and PPH.

## Methods

### Study Design and Population

This retrospective cohort study was conducted at the Shanghai First Maternity and Infant Hospital, which is one of the largest tertiary maternity hospitals in Shanghai, China, with over 20,000 deliveries annually. No special informed consent was obtained, and the hospital ethics committee approved the study (registration number: KS20279). Between August 2013 and July 2020, a total of 3,395 twin gestations were delivered *via* cesarean section in the hospital. We included patients who delivered after 22 weeks' gestation and had complete medical records available for review. The exclusion criteria included cases where: (1) one fetus died *in utero*; (2) a combined delivery was performed (vaginal delivery of the first twin followed by cesarean section of the second twin); and (3) fetal structural anomalies or aneuploidy were present. Finally, 3,180 twin gestations were analyzed.

### Data Collection

We reviewed the obstetric records of all women with twin pregnancies undergoing cesarean section. We extracted the demographic information of the participants from a computerized database and double-checked the data. Data on potential risk factors for PPH were collected: maternal age, gravidity, parity, ART use, gestational age at delivery, pre-pregnancy body mass index (BMI), previous cesarean section, placenta previa, uterine myoma, hypertensive disorders, placental abruption, placenta accreta/increta/percreta, low-lying placenta, hematocrit, premature rupture of membranes, emergency cesarean delivery, age at menarche, prenatal fever, and thrombocytopenia. We also collected data on unique features of the twins, such as the sex of the twins, the individual and combined birth weight of the twins, and twin chorionicity.

### Definition of Abnormal Fetal Growth

The definition of FGR in twin pregnancies is inconsistent in the literature, and two separate criteria have been applied: at least one fetus with a birth weight < the 10th percentile (4, 26, 27), or at least one fetus with a birth weight < the 3rd percentile (28–30). Based on the definition of selective fetal growth restriction (sFGR) in the recent Delphi Consensus Statement, we adapted the antenatal criteria of one of the following: (1) one twin with a birth weight < the 10th centile and a birth weight discordance of >25%, or (2) the solitary criterion of one twin with a birth weight < the 3rd percentile (28, 30, 31). Singleton reference charts were used to assess birth weight centiles (28). Growth discordance was defined as a twin birth weight difference of ≥20% (twin birth weight difference = (large fetal body weight – small fetal body weight) / large fetal body weight × 100%) (32, 33).

### PPH Diagnosis

The primary outcome was PPH, which was identified in our dataset using the International Classification of Diseases, Eleventh Revision, Clinical Modification (ICD-11-CM) diagnosis codes JA43.1. Secondary outcomes included PPH with severe maternal morbidity (SMM) and PPH with hemostatic interventions. We used a combination of visual estimation and quantitative blood loss (QBL) measurement to qualify blood loss (34, 35). PPH was defined as blood loss of ≥1,000 ml following delivery (36, 37). PPH with SMM was a composite outcome defined as PPH associated with any of the following: transfusion of ≥4 units of packed red blood cells, hysterectomy, return to the operating room for exploratory laparotomy, and intensive care unit admission for invasive monitoring or treatment. PPH with hemostatic interventions was defined as PPH associated with uterine artery ligation, intrauterine balloon tamponade, or uterine compression suture.

### Statistical Analysis

Statistical analysis was performed using SPSS (version 16.0, SPSS Inc., Chicago, IL, USA). Continuous variables with normal distributions are presented as mean ± standard deviation (SD), while categorical data are presented as percentages (%). Parametric *t*-tests and χ^2^ tests were used to compare the differences in demographic characteristics between women with PPH and women without PPH.

The associations of FGR or twin growth discordance with the risk of PPH were estimated using a multivariable logistic regression analysis, with odds ratios (ORs) corresponding to 95% confidence intervals (CIs) as risk measures. We adjusted for the following covariates that have been shown to be associated with PPH (17–25, 38): maternal age, pre-pregnancy BMI, gravidity, parity, gestational age at delivery, age at menarche, placental abruption, placenta previa, placenta accreta, placenta increta, low-lying placenta, premature rupture of membranes, hematocrit of <30%, reduced platelet count (<70,000/μL), gestational hypertension, the use of ART, emergency operation, previous cesarean section, gestational diabetes mellitus, uterine myoma, prenatal fever, and twin chorionicity.

In order to exclude the impact of placenta accreta and increta on the association between abnormal fetal growth and PPH, we conducted a sensitivity analysis by restricting all logistic regression analyses to participants without placenta accreta or increta. Further, to investigate the potential modification effect of twin chorionicity, we stratified the study population by twin chorionicity and assessed the association of abnormal fetal growth with PPH in dichorionic and monochorionic twins separately. All analyses were bilateral, and statistical significance was set at *P* < 0.05.

## Results

### Characteristics of the Study Participants

Altogether, 3,180 twin pregnancies were included in the final analysis. The overall incidence of PPH in this cohort was 4.3% (*n* = 138). The incidence of PPH with SMM and PPH with obstetric surgical procedures was 3.3% and 3.6%, respectively. Table 1 presents the participants' general characteristics. Women with PPH were significantly older than those without PPH (32.2 ± 3.8 vs. 31.5 ± 3.9, respectively, *P* = 0.043). The incidence of hypertensive disorders, hematocrit of <30%, and the use of ART were significantly higher in women with PPH than in women without PPH (*P* < 0.001, *P* = 0.021, *P* < 0.001, respectively). Moreover, the incidence of an abnormal placenta, such as placental abruption, placenta previa, placenta accreta, and placenta increta, was significantly different between the two groups (All values were *P* < 0.001).

**Table 1:** Baseline characteristics of twin pregnancies complicated by PPH compared to non-PPH twin pregnancies.

**Characteristics**	**PPH (*N*=138)**	**Non-PPH (*N*=3,042)**	***P*-value^a^**
**Demographic**			
Maternal age (years)	32.2 ± 3.8	31.5 ± 3.9	0.043
Parity			
Nulliparous	121 (87.7%)	2,530 (83.2%)	0.164
Multiparous	17 (12.3%)	512 (16.8%)	
Gravidity			
<4	124 (89.9%)	2,804 (92.2%)	0.323
≥4	14 (10.1%)	238 (7.8%)	
Age at menarche			
≤ 12	20 (14.5%)	470 (15.5%)	0.949
13–14	90 (65.2%)	1,973 (64.9%)	
≥15	28 (20.3%)	599 (19.6%)	
Pre-pregnancy BMI (kg/m^2^)			
<18.5	8 (5.8%)	222 (7.3%)	0.647
18.5–24.9	109 (79.0%)	2,301 (75.6%)	
≥25	21 (15.2%)	519 (17.1%)	
Weight gain during pregnancy pregnancy	15.1 ± 5.8	14.4 ± 5.2	0.169
The use of ART	111 (80.4%)	1,893 (62.2%)	<0.001^**^
Pregnancy-related variables			
GDM	21 (15.2%)	523 (17.2%)	0.547
Hypertensive disorders	46 (33.3%)	542 (17.8%)	<0.001^**^
Placental abruption	4 (2.9%)	27 (0.9%)	<0.001^**^
Placenta previa	20 (14.5%)	24 (0.8%)	<0.001^**^
Placenta accreta	49 (35.5%)	221 (7.3%)	<0.001^**^
Placenta increta	8 (5.8%)	13 (0.4%)	<0.001^**^
Placenta Low-lying	9 (6.5%)	102 (3.4%)	0.056
Surgery-related variables			
PLT <70	0 (0.0%)	10 (0.3%)	>0.999
HCT <30%	24 (17.4%)	336 (11.0%)	0.021^*^
Emergency operation	45 (32.6%)	879 (28.9%)	0.347
General anesthesia	8 (5.8%)	86 (2.8%)	0.064

*All data are presented as the mean ± standard deviation or n (%)*.

a *P values were calculated by t-tests and χ^2^ tests*.

* *P < 0.05*,

** *P < 0.01*.

### Associations Between Abnormal Fetal Growth and Risk of PPH

The associations of FGR or twin growth discordance with the risk of PPH are shown in Table 2. In general, both FGR and sFGR were not associated with PPH, while twin growth discordance was significantly associated with an increased risk of PPH. In the adjusted model, compared to mothers of concordant growth twins, the odds of PPH increased by 62% (OR = 1.62, 95% CI, 1.05–2.51) for mothers of discordant twins. The co-presence of twin growth discordance and FGR was also significantly associated with an increased risk of PPH.

**Table 2:** Logistic regression analysis for the associations between twin growth discordance, fetal growth restriction (FGR), and risk of PPH.

	**Crude model**		**Adjusted model**	
	**OR (95% CI)**	***p-*value**	**OR (95% CI)**	***P*-value^**a**^**
FGR in at least one twin (BW <10th)	0.71 (0.50, 1.01)	0.059	0.75 (0.51, 1.11)	0.151
FGR in at least one twin (BW <3rd)	1.02 (0.68, 1.53)	0.926	1.20 (0.78, 1.86)	0.408
sFGR	0.98 (0.65, 1.47)	0.918	1.13 (0.73, 1.75)	0.578
Twin growth discordance	1.64 (1.10, 2.45)	0.015*	1.62 (1.05, 2.51)	0.031*
Twin growth discordance plus FGR(BW <10th)	1.61 (1.06, 2.44)	0.025*	1.71 (1.08, 2.70)	0.022*
Twin growth discordance plus FGR(BW <3rd)	1.65 (1.05, 2.58)	0.028*	1.98 (1.21, 3.25)	0.006*

*Adjustment for Maternal age, pre-pregnancy BMI, gravidity, parity, gestational age at delivery, age at menarche, placental abruption, placenta previa, placenta accrete, placenta increta, placenta low-lying, premature rupture of membranes, HCT <30%, reduced platelet count (<70,000/μL), gestational hypertension, the use of ART, emergency operation, previous C-S, gestational diabetes mellitus, uterine myoma, prenatal fever, and twin chorionicity*.

### Association Between Fetal Growth Anomalies and Secondary Outcomes

Table 3 presents the association of abnormal fetal growth with the risk of two secondary outcomes: PPH with SMM and PPH with hemostatic interventions. Similar to the results of PPH, both twin growth discordance and co-presence of twin growth discordance with FGR were associated with an increased risk of PPH with hemostatic interventions. In the adjusted model, compared to mothers of concordant growth twins, the odds of PPH with hemostatic interventions increased by 62% (OR = 1.62, 95% CI, 1.00–2.63) in mothers of discordant twins. For PPH with SMM, only the co-presence of twin growth discordance with FGR (birth weight <3rd percentile) was associated with an increased risk of PPH with SMM (OR = 2.07; 95% CI, 1.17–3.68).

**Table 3:** The Associations between twin growth discordance, fetal growth restriction (FGR), and secondary outcomes.

	**PPH with SMM**		**PPH with hemostatic interventions**	
	**OR (95% CI)**	***p*-value**	**OR (95% CI)**	***P*-value**
FGR in at least one twin (BW <10th)	0.73 (0.47, 1.14)	0.730	0.75 (0.49, 1.14)	0.177
FGR in at least one twin (BW <3rd)	1.23 (0.74, 2.04)	0.429	1.26 (0.78, 2.03)	0.345
sFGR	1.15 (0.69, 1.91)	0.584	1.18 (0.73, 1.91)	0.493
Twin growth discordance	1.51 (0.90, 2.51)	0.117	1.62 (1.00, 2.63)	0.049^*^
Twin growth discordance plus FGR (BW <10th)	1.63 (0.95, 2.79)	0.074	1.70 (1.03, 2.80)	0.040^*^
Twin growth discordance plus FGR (BW <3rd)	2.07 (1.17, 3.68)	0.013^*^	2.02 (1.18, 3.47)	0.011^*^

*Adjustment for Maternal age, pre-pregnancy BMI, gravidity, parity, gestational age at delivery, age at menarche, placental abruption, placenta previa, placental accrete, placental increta, placenta low-lying, premature rupture of membranes, HCT <30%, reduced platelet count (<70,000/μL), gestational hypertension, the use of ART, emergency operation, previous C-S, gestational diabetes mellitus, uterine myoma, prenatal fever, and twin chorionicity*.

* *P < 0.05*.

### Sensitivity Analysis

In the sensitivity analysis, when restricting the analysis to participating women without placenta accreta or placenta increta, the association between twin growth discordance or co-presence of twin growth discordance with FGR and PPH risk did not appreciably change. However, the 95% CIs for ORs became wider and the results were no longer statistically significant and the results were no longer statistically significant owing to a reduction in the sample size (Table 4).

**Table 4:** The Association between intertwin growth discordance, fetal growth restriction (FGR), and PPH among participants without placenta accrete or placenta increta.

	**OR (95% CI)**	***P*-value**
FGR in at least one twin (BW <10th)	0.69 (0.44, 1.09)	0.691
FGR in at least one twin (BW <3rd)	1.01 (0.60, 1.71)	0.967
sFGR	0.95 (0.56, 1.60)	0.837
Twin growth discordance	1.59 (0.94, 2.69)	0.082
Twin growth discordance plus FGR (BW <10th)	1.62 (0.94, 2.79)	0.083
Twin growth discordance plus FGR (BW <3rd)	1.70 (0.95, 3.06)	0.074

*Adjustment for Maternal age, pre-pregnancy BMI, gravidity, parity, gestational age at delivery, age at menarche, placental abruption, placenta previa, placenta low-lying, premature rupture of membranes, HCT <30%, reduced platelet count (<70,000/μL), gestational hypertension, the use of ART, emergency operation, previous C-S, gestational diabetes mellitus, uterine myoma, prenatal fever, and twin chorionicity*.

We further stratified the participants by twin chorionicity and estimated the association of abnormal fetal growth with PPH in dichorionic and monochorionic twins separately. As shown in Table 5, among mothers of dichorionic twins, twin growth discordance and co-presence of twin growth discordance with FGR was significantly associated with an increased risk of PPH. However, among mothers of monochorionic twins, no significance was found.

**Table 5:** The Association between intertwin growth discordance, fetal growth restriction, and PPH stratified by chorionicity.

	**DC**		**MC**	
	**OR (95% CI)**	***P*-value**	**OR (95% CI)**	***P*-value**
FGR in at least one twin (BW <10th)	0.84 (0.55, 1.29)	0.422	0.44 (0.17, 1.12)	0.085
FGR in at least one twin (BW <3rd)	1.35 (0.82, 2.22)	0.236	0.73 (0.28, 1.91)	0.519
sFGR	1.26 (0.77, 2.06)	0.366	0.70 (0.27, 1.83)	0.467
Twin growth discordance	1.71 (1.04, 2.82)	0.036^*^	1.61 (0.61, 4.23)	0.333
Twin growth discordance plus FGR(BW <10th)	1.90 (1.13, 3.21)	0.016^*^	1.36 (0.50, 3.70)	0.553
Twin growth discordance plus FGR(BW <3rd)	2.48 (1.41, 4.38)	0.002^*^	1.22 (0.42, 3.53)	0.714

*Adjustment for Maternal age, pre-pregnancy BMI, gravidity, parity, gestational age at delivery, age at menarche, placental abruption, placenta previa, placenta accrete, placenta increta, placenta low-lying, premature rupture of membranes, HCT <30%, reduced platelet count (<70,000/μL), gestational hypertension, the use of ART, emergency operation, previous C-S, gestational diabetes mellitus, uterine myoma, and prenatal fever*.

*DC, dichorionic; MC, monochorionic*;

* *P < 0.05*.

## Discussion

### Principal Findings

Our findings demonstrated that twin growth discordance or twin growth discordance complicated by FGR was associated with an increased risk of PPH, and the associations were much more evident in mothers of dichorionic twins than in mothers of monochorionic twins. However, we found no association between FGR and PPH. Our findings suggest that twin growth discordance is an independent risk factor for PPH.

### Interpretation of the Findings

PPH is one of the leading causes of maternal morbidity and mortality worldwide (39–42). Over the past few decades, many studies have focused on the predictors of PPH, and several factors have been reported to be associated with PPH (17–25). Several maternal risk-assessment tools, such as the California Maternal Quality Care Collaborative, the Association of Women's Health, Obstetric and Neonatal Nurses, and the New York Safety Bundle for Obstetric Hemorrhage, have also been developed to assess the risk of PPH (43). Compared to singleton gestations, PPH is more common in twin pregnancies, as uterine overdistention may impair the contraction of uterine muscles and increase the incidence of uterine atony after delivery (44). To date, no study has examined the association between FGR and PPH in twins. However, in singleton pregnancies, a study has reported that women with pregnancies complicated by both preeclampsia and FGR are more likely to experience abruption, have a higher rate of cesarean delivery, and undergo cesarean delivery for fetal heart rate abnormalities, but not for PPH (45).

Growth discordance is a proprietary feature of twin pregnancies, and it is one of the major determinants of perinatal outcomes in twin pregnancies (11, 12). Here, the overall incidence rate was approximately 16.9%, which is consistent with the results of previous studies (4, 11, 32). Higher degrees of discordance are known to be associated with perinatal mortality and morbidity (11, 13–15). A study has shown that a within-pair birth weight difference of >25% increases the risk of both intra-uterine death and neonatal death (13). However, whether there is a correlation between twin growth discordance and PPH remains unknown. To the best of our knowledge, this is the first report on the association between growth discordance and PPH in women with twin pregnancies.

The underlying mechanism between twin growth discordance and PPH is unclear. A potential mechanism may involve the notion that growth discordance increases the risk of preeclampsia or hypertension (28, 32), which are important risk factors for PPH (18, 22, 24). Interestingly, in our study, after adjusting for hypertensive disorders, we found that the association between twin growth discordance and PPH remained significant. Another possible explanation for the observed association is that twin growth discordance and PPH may have some common risk factors. Known risk factors for twin growth discordance are advanced maternal age and parity, conception by ART, maternal hypertensive disorders in pregnancy, opposite sex of twin fetuses, and exposure to air pollution during pregnancy (46–48). These can be categorized as maternal, fetal, placental, or environmental risk factors. Among these factors, advanced maternal age and parity, conception by ART, and maternal hypertensive disorders are also risk factors for PPH (17, 19, 20, 22, 24, 49). Other pathophysiological mechanisms include histological placenta and inflammatory responses (32, 50). Frequently, histological abnormalities in placentas were found among smaller twins of birth weight discordant pairs (50). Oxidative stress and apoptosis in the placenta may result from the increased demand imposed by twins and from placental ischemia/hypoxia, leading to growth discordance (51). Additionally, various active molecules released into the maternal circulation due to placental ischemia/hypoxia causes vasoconstriction and an increase in blood pressure, and this is due to generalized endothelial dysfunction and an exaggerated inflammatory response (32).

We also found that the relationship between growth discordance and PPH existed only among mothers of dichorionic twins but not among those with monochorionic twins. Fetal growth during gestation is generally determined by fetal, maternal, and uterine placental factors. Discordant growth among dichorionic twins may be due to the poor ability of the smaller twin to realize its growth potential, which commonly suggests a pathological condition, such as placental vascular dysfunction. For monochorionic twins, the cause of discordant growth is related to unequal distribution of uteroplacental blood flow between two fetuses, abnormal umbilical cord insertion site, and critically by twin-to-twin transfusion syndrome, while factors related to placental histology are rare (52–55). In a prospective study of 1,001 twin pairs, an association between abnormalities in placental histology and birth weight discordance was found in dichorionic twins, but not in monochorionic twins (50). Similarly, another study showed that excess placental apoptosis and changes in the synthesis of various trophoblastic proteins were found in discordant dichorionic twins (51). The incidence of preeclampsia in women with dichorionic pregnancies is much higher than that in women with monochorionic twins (56, 57). Qiao et al. reported that twin growth discordance was associated with a high risk of preeclampsia among women with dichorionic twins (32). All of the above factors may partially explain the relationship between growth discordance and PPH in the dichorionic twin group. Compared to singleton gestation, twin gestation increased the risk of placenta accreta spectrum (including placenta accreta, increta, and percreta) independent of measured risk factors (58). Besides, in twin pregnancies, the assisted reproductive technology is independently associated with placenta accreta spectrum (59). Based on the above reasons, the incidence of placenta accreta spectrum is higher in twins. This may explain why the association between twin growth discordance or twin growth discordance complicated by FGR and PPH was not apparent when we excluded these twins with placenta accreta spectrum.

### Strengths and Limitations

This study is the first report on the relationship between growth discordance and PPH. Another strength of our study is the relatively large sample size from a single center. However, our study has several limitations. First, it only included twin pregnancies delivered *via* cesarean section. Due to physician counseling and maternal requirements for selective cesarean section to avoid combined delivery, the cesarean delivery rate of twin pregnancies has increased dramatically (60–62). In our center, nearly 90% of women with twin pregnancies undergo a cesarean section. During a cesarean section, the amniotic fluid is absorbed, and another aspirator is used to absorb blood, and this accurately measures the estimated amount of blood loss; however, it is impossible to accurately estimate the blood loss in vaginal deliveries. Second, due to the lack of unified international standard for the diagnosis of FGR in twins, we used the singleton birth weight standards as the diagnostic criteria of FGR. Recently, twin-specific charts were recommended when evaluating fetal growth in twin pregnancies in some studies (28, 30, 63). However, pathological growth tracks and at-risk fetuses may be obscured by twin-specific growth charts. Finally, although we adjusted for various confounders, some unmeasured confounders may invalidate the association between growth discordance and PPH.

## Conclusions

We found that twin growth discordance, rather than FGR, was associated with an increased risk of PPH. Our findings suggest that twin growth discordance is an independent risk factor for PPH. This signals obstetricians and midwives to implement early preventative strategies for PPH among mothers of growth discordant twins.

## Data Availability Statement

The raw data supporting the conclusions of this article will be made available by the authors, without undue reservation.

## Ethics Statement

The studies involving human participants were reviewed and approved by Ethics Committee of Shanghai First Maternity and Infant Hospital, School of Medicine, Tongji University. Written informed consent for participation was not required for this study in accordance with the national legislation and the institutional requirements.

## Author Contributions

XC, YL, and SZ: full access to all of the data in the study, take responsibility for the integrity of the data, the accuracy of the data analysis, and drafting of the manuscript. XC and ZX: concept and design. XC, YL, SZ, XQ, and ZX: critical revision of the manuscript for important intellectual content. QZ and ZX: statistical analysis. YL, QZ, and XQ: administrative, technical, or material support. ZX and ZL: supervision. All authors: acquisition, analysis, or interpretation of data.

## Funding

This work was supported by the grants of Pudong Health Committee of Shanghai (PW2021D-01 and PW2020D-13).

## Conflict of Interest

The authors declare that the research was conducted in the absence of any commercial or financial relationships that could be construed as a potential conflict of interest.

## Publisher's Note

All claims expressed in this article are solely those of the authors and do not necessarily represent those of their affiliated organizations, or those of the publisher, the editors and the reviewers. Any product that may be evaluated in this article, or claim that may be made by its manufacturer, is not guaranteed or endorsed by the publisher.

## Abbreviations

ART: assisted reproductive technology
FGR: fetal growth restriction
PPH: Postpartum hemorrhage
SMM: severe maternal morbidity
OR: odds ratio
CI: confidence interval
sFGR: selective fetal growth restriction.
